# Non-convulsive epiletic seizure after electroconvulsive therapy session

**DOI:** 10.1192/j.eurpsy.2023.2169

**Published:** 2023-07-19

**Authors:** L. Llovera García, L. Veiga Gil, L. Lopez Unzue, A. Corrales Rodriguez, A. Ballesteros Prados, J. Yoldi Murillo, J. M. Lopez Ilundain

**Affiliations:** 1 Psychiatry; 2 Anaesthesiology, Navarra’s University Hospital, Pamplona; 3 Psychiatry, Osakidetza, Vitoria, Spain

## Abstract

**Introduction:**

Electroconvulsive therapy (ECT) is a procedure performed under general anaesthesia involving triggering an intentional brief seizure through small electrical currents through the brain. The anaesthetic depth should be adequate prior to shock and measured with BIS, a processed electroencephalogram (EEG) monitor. Adjusting the hypnotic dose allows to decrease the ictal threshold and thus improve the response to treatment and decrease side effects.

**Objectives:**

Our goal is detecting elements such as spontaneous epileptiform activity after ECT without tonic-clonic activity with the spectral density matrix (SDM).

**Methods:**

Our patient: an 87-year-old woman, diagnosed with F20.2 catatonic schizophrenia and under antipsychotic treatment since her youth. She has required multiple hospital admissions due to psychopathological decompensations until starting monthly maintenance ECT sessions in 2014. Since then she no new hospital admissions have been required.

**Results:**

Images 1D and 1E shows the SDM, a spectrogram of the EEG. The X axis show time (minutes), the Y axis shows the frequency (Hz) and the Z axis shows the energy or intensity of that electrical activity in the frequency bands replaced by colors: warm colors (red) reflecting high intensity electroencephalographic activity and cool colors (yellow, blue and green), low activity. Images 2 and 3’s EDM shows spontaneous epileptiform activity after electroconvulsive therapy without tonic-clonic activity. We observed an initial EDM of an awake patient, with warm colours in practically all frequency bands, including the beta band (13-30 Hz), characteristic of waking states. Around 9:50 anaesthetic induction occurs, activity increases in slow frequencies (red colours in alpha, theta and delta), plus an increase of cold colours in beta, reflecting the disappearance of brain activity in that frequency. The asterisk reflects the EEG response to the electrical discharge, followed by a postcritical state with brain activity exclusively in slow waves and high amplitude (delta and some theta) and absence of activity in other frequencies (blue colour in the beta and alpha bands) around 9:57. At about 10:00 there is an abrupt appearance of high intensity brain activity (warm colours) in beta and alpha and delta, mainly, reflecting spontaneous epileptiform activity after treatment and clinically reflected as a patient absent and disconnected from the environment, but without tonic-clonic activity. New postcritical state in which blue colour predominates, reflecting little brain activity, and warmer colours reappear in all frequency bands, including beta, reflecting the progressive recovery of wakefulness.

**Image:**

**Figure:**
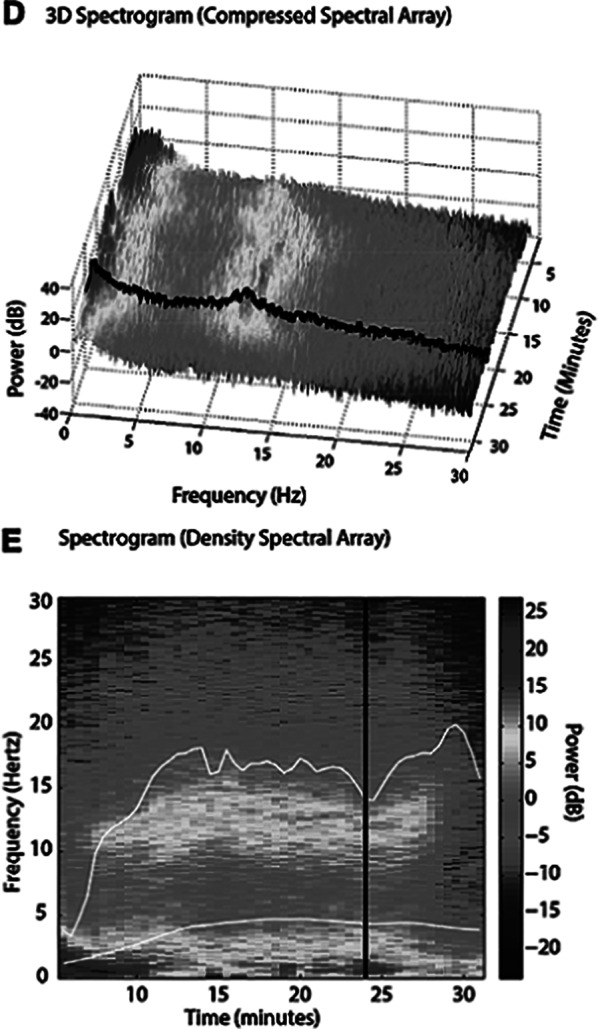

**Image 2:**

**Figure:**
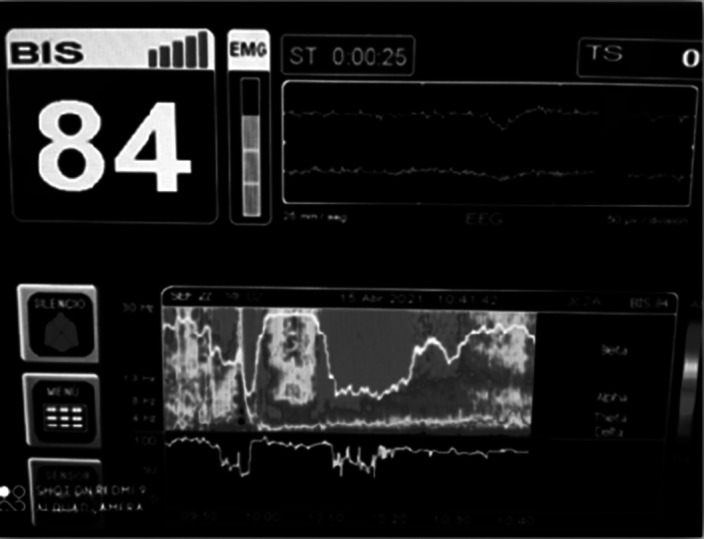

**Image 3:**

**Figure:**
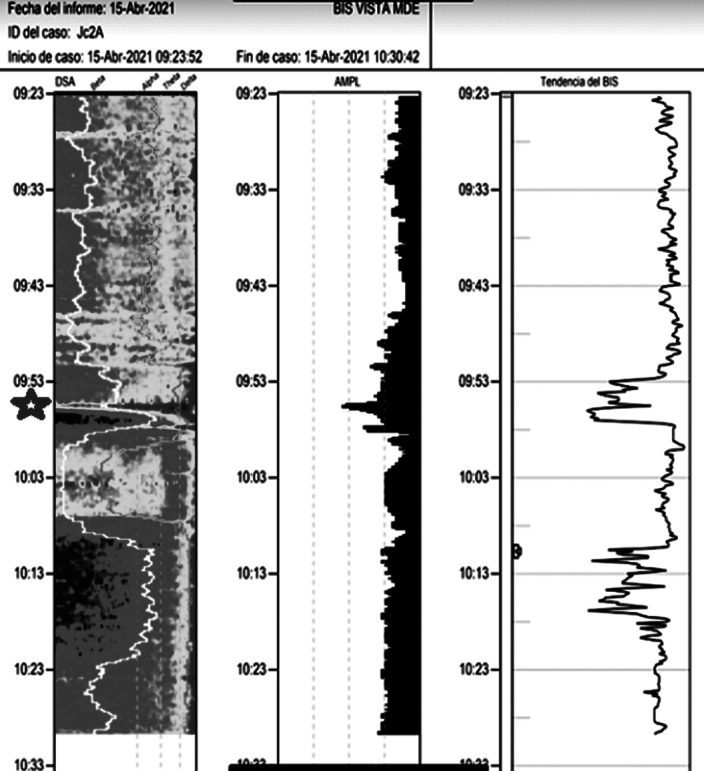

**Conclusions:**

Psychiatric pathology can be reflected in the SDM, which allows to observe changes in the EEG, correcting the electrical stimulus of the shock and the dose of anesthetic appropriate to the patient to trigger an intentional brief seizure under general anesthesia.

**Disclosure of Interest:**

None Declared

